# Sirt6 alters adult hippocampal neurogenesis

**DOI:** 10.1371/journal.pone.0179681

**Published:** 2017-06-23

**Authors:** Eitan Okun, Daniel Marton, Daniel Cohen, Kathleen Griffioen, Yariv Kanfi, Tomer Illouz, Ravit Madar, Haim Y. Cohen

**Affiliations:** 1 The Mina and Everard Goodman faculty of Life sciences, Bar Ilan University, Ramat Gan, Israel; 2 The Leslie and Susan Gonda Multidisciplinary Brain Research Center, Bar-Ilan University, Ramat Gan, Israel; 3 The Paul Feder Laboratory on Alzheimer's disease research, Bar-Ilan University, Ramat Gan, Israel; 4 Department of Biology and Chemistry, Liberty University, Lynchburg, VA, United States of America; University of Nebraska Medical Center, UNITED STATES

## Abstract

Sirtuins are pleiotropic NAD^+^ dependent histone deacetylases involved in metabolism, DNA damage repair, inflammation and stress resistance. SIRT6, a member of the sirtuin family, regulates the process of normal aging and increases the lifespan of male mice over-expressing Sirt6 by 15%. Neurogenesis, the formation of new neurons within the hippocampus of adult mammals, involves several complex stages including stem cell proliferation, differentiation, migration and network integration. During aging, the number of newly generated neurons continuously declines, and this is correlated with a decline in neuronal plasticity and cognitive behavior. In this study we investigated the involvement of SIRT6 in adult hippocampal neurogenesis. Mice over-expressing Sirt6 exhibit increased numbers of young neurons and decreased numbers of mature neurons, without affecting glial differentiation. This implies of an involvement of SIRT6 in neuronal differentiation and maturation within the hippocampus. This work adds to the expanding body of knowledge on the regulatory mechanisms underlying adult hippocampal neurogenesis, and describes novel roles for SIRT6 as a regulator of cell fate during adult hippocampal neurogenesis.

## Introduction

Neurogenesis is a process by which new neurons are generated from neural stem cells and committed neuronal progenitor cells (NPC). Neurogenesis is most pronounced during embryonic development and is responsible for populating the growing brain with neurons. Neurogenesis also occurs in the adult brain, and normally occurs throughout adult murine life in the sub-ventricular zone (SVZ) bordering the lateral ventricles and in the dentate gyrus (DG) of the hippocampal sub-granular zone (SGZ) [1]. In the adult SGZ, proliferating radial and non-radial precursors give rise to intermediate progenitors, which in turn generate neuroblasts. Further differentiation of these neuroblasts produces immature neurons that migrate into the inner granule cell layer and differentiate into dentate granule cells within the DG of the hippocampus [1]. Within days, newborn neurons extend dendrites toward the molecular layer and project axons towards the CA3 sub-region of the hippocampus, where they integrate into the existing neuronal circuitry [1]. Synaptically-connected newborn neurons exhibit distinct hyper-excitability and enhanced synaptic plasticity. These properties may allow newly integrated adult-born neurons to make unique contribution to information processing [1]. Thus, neurogenesis from adult NPCs is thought to contribute to the continuous maintenance and plasticity of these neuronal networks.

Sirtuins are pleiotropic NAD^+^ dependent histone deacetylases involved in metabolism, DNA damage repair, inflammation and stress resistance. Sirtuins can alter adult NPC proliferation by altering the oxidizing state of the environment. Whereas oxidizing conditions favor differentiation into astrocytes, reducing conditions favor differentiation into neurons [2]. Activating SIRT1 in NPCs using resveratrol mimics oxidizing conditions and increases the differentiation of NPCs towards astrocytes [2]. Interestingly, resveratrol exerts a dose dependent effect on neurogenesis with lower concentrations promoting neurogenesis [3] and higher doses inhibiting neurogenesis [4]. Further, in Sirt1 knockout (KO) mice, as well as in brain-specific and inducible stem cell-specific conditional KO mice, hippocampal neurogenesis is increased [4]. This suggests that Sirtuins have important roles in the regulation of neuronal cell fate.

SIRT6, a member of the sirtuin family, regulates the process of normal aging and increases the lifespan of male mice over-expressing Sirt6 by 15% [5]. Aging is correlated with a continuous decline in the number of adult NPCs, reduced DG neurogenesis capacity, and impaired cognitive function [6]. Interestingly, such impairments can be ameliorated through interventions such as physical exercise and calorie restriction (CR) [6–8]. In both exercise and CR, the AMP/ATP ratio is increased and AMP-activated protein kinase (AMPK) is activated [9, 10]. As AMPK is an activator of Nicotinamide phosphoribosyltransferase (NAMPT), the rate-limiting enzyme in NAD^+^ biosynthesis, the overall NAD^+^ turnover under exercise and CR is increased [11, 12]. The dependence on NAD^+^ as a cofactor offers a link between Sirtuins and the beneficial effects exerted by exercise and CR. SIRT6, a member of the sirtuin family is expressed in the nucleus of cells in the mouse hippocampal formation [13], positively regulates AMPK activation levels [14, 15], embryonic stem cell proliferation [16] and differentiation of several cell types such as bone marrow, chondrocytes and vascular smooth muscle [17–19]. However, whether Sirt6 alters adult hippocampal neurogenesis is unknown.

Because Sirt6 is expressed in the hippocampus and is implicated in embryonic neurogenesis, we hypothesized that Sirt6 also alters adult hippocampal neurogenesis. Here we show that while NPC proliferation is unaffected, Sirt6-OE decreases the number of mature neurons in the hippocampus, and alters the distribution of neural precursors differentiating into neurons. Our study points to the complex role played by Sirt6 in regulating adult hippocampal neurogenesis and expands the current understanding of adult hippocampal neurogenesis.

## Materials and methods

### Animals

Mice with a heterozygous Sirt6 overexpression (OE) and their wildtype littermates were generated by backcrossing CB6 Sirt6-OE mice [5] with C57bl/6H mice for 20 generations. The initial CB6 SIRT6-OE mice were generated by isolating Sirt6 mRNA from C57BL/6J male mouse brain tissue. The complete mouse SIRT6 cDNA was cloned into a pCAGGS plasmid attached to CMV enhancer and chicken β-actin promoter [20]. Linearized SIRT6 construct was microinjected into CB6/F1 zygotes [21]. Following this, Sirt6 OE mice as well as their wild type (WT) littermates were maintained at a 12- light/12- dark cycle with food and water provided ad libitum. All experiments were performed using 2-3-months old male mice. Interventions in mice performed in this study include intraperitoneal injections of Bromo-2´-Deoxyuridine (BrDU). Following injections, mice were euthanized prior to culling, thus the mice were not exposed to suffering which required analgesics. All experiments in study received specific approval and were conducted in accordance with the Bar Ilan University’s Institutional Animal Care and Use Committee (IACUC).

### 5-Bromo-2´-Deoxyuridine incorporation for the assessment of neurogenesis

5-Bromo-2´-Deoxyuridine (BrdU; MP biomedicals) was dissolved in 0.9% saline and sterile filtered at 0.2μm. All mice were injected with a BrdU dose of 100mg/kg body weight per injection to label dividing cells. Three experimental groups of mice were used in this study to quantify hippocampal progenitor cells, young and adult neurons; Experimental group 1 (n = 10 WT, 8 Sirt6-OE) was injected 3 times at 8 hour intervals and euthanized 8 hours after the last injection to assess the neurogenic niche. Experimental group 2 (n = 8 WT, 10 Sirt6-OE) was injected for 2 days at 8 hour intervals and euthanized 7 days after the last injection to assess early neurogenesis. Experimental group 3 (n = 9 WT, 8 Sirt6-OE) was injected for 5 days at 12 hour intervals and euthanized 5 weeks after the last injection to assess late neurogenesis (see Table 1). Following injections, mice were returned to their home cage. Specified intervals between BrdU pulses and animal culling were chosen according to known maturation marker expression timelines [22].

**Table 1:** Experimental design.

Experiment Group	Neurogenesis stage	Cell type	BrdU injection scheme (100 mg/kg)	Number of mice	Time of animal sacrifice	IHC marker (co-localized with BrdU)
1	Proliferation	NPC	3 injections, 8 hours interval	WT = 10, Sirt6-OE = 8	8 hours after last injection	Sox2
2	Differentiation	Immature neuron	6 injections, 8 hours interval	WT = 8, Sirt6-OE = 10	5 days after last injection	DCX
3	Maturation	Mature neuron	10 injections, 12 hours interval	WT = 9, Sirt6-OE = 8	4 weeks after last injection	NeuN

Each of the 3 experimental groups, consisting of WT and Sirt6-OE mice, were assigned to investigate different stages of the neuronal maturation process. Experimental group 1 (n = 10 WT, 8 Sirt6-OE) was injected with BrdU (100mg/kg) 3 times at 8 hour intervals and sacrificed 8 hours after the last injection for co-immunostaining of the NPC marker Sox2 with BrdU. This corresponds to the progenitor proliferation stage. Experimental group 2 (n = 8 WT, 10 Sirt6-OE) was injected with BrdU (100mg/kg) for 2 days at 8 hour intervals and sacrificed 5 days after the last injection for co-immunostaining of the immature neuron marker DCX with BrdU. This corresponds to the neuronal differentiation stage. Experimental group 3 (n = 9 WT, 8 Sirt6-OE) was injected with BrdU (100mg/kg) for 5 days at 12 hour intervals and sacrificed 4 weeks after the last injection for co-immunostaining of mature neuron marker NeuN with BrdU. This corresponds to the neuronal maturation stage.

### Immunofluorescence

To perform immunofluorescence on brain slices, mice were subjected to a common perfusion/fixation protocol [23]. Briefly, Mice were anesthetized using Ketamine / Xylazine (10mg/kg, and 10mg/kg respectively) and perfused transcardially with cold 4% paraformaldehyde (PFA) in 0.1 M PBS [23]. Brains were removed and post fixed in 4% PFA overnight and then sequentially cryoprotected in 20% and 30% sucrose in 0.1 M PBS. Brains were then sectioned 40 μm thick on a freezing microtome in the coronal plane. All immunohistochemistry was completed as free-floating sections and mounted on gelatin-coated slides for analysis. For 5-Bromo-2´-Deoxyuridine (BrdU) staining, sections were first immersed in a 2N HCl for 30 min at 37°C, followed by 0.1 M borate buffer (pH 8.5) for 10 min at room temperature. Sections were then washed six times in 0.1% Triton X-100 in Phosphate-buffered saline (PBS) for a total of 30 min. Nonspecific binding was blocked with 20% normal horse serum and 0.1% Triton X-100 in PBS for 1 hour. Primary antibodies used for staining were: rat anti-BrdU (1:1000; serotec, OBT0030), rabbit anti-SOX2 (1:1000; abcam, ab97959), goat anti-Doublecortin (DCX) (1:250; Santa Cruz, sc-8066), rabbit anti-S100β (1:7,500; Novus, NB110-57478) and mouse anti-NeuN (1:10,000; Millipore, MAB377). All antibodies were diluted in PBS supplemented with 0.1% Triton-X-100 with 2% horse serum. Following 72 primary antibody incubation, sections were washed three times in 0.1% Triton X-100 in PBS for a total of 15 min. Sections were subsequently incubated with a fluorescent-tagged secondary antibody (Alexa-488 or Alexa-568 1:1,000; Invitrogen), diluted in PBS supplemented with 0.1% Triton X-100 for 1 at room temperature.

### Assessment of neurogenesis, glial and neuronal cell numbers using stereology

The hippocampus and DG were outlined based on an atlas of the mouse brain [24]. Quantification of stained cells was evaluated by stereological counts using the optical dissector method [25]. Optical fractionator sampling was carried out on a Leica DM6000 microscope (Leica Microsystems) coupled to a controller module and a high-sensitivity 3CCD video camera system (MBF Biosciences), and an Intel Xeon workstation (Intel). Sampling was implemented using the Stereo Investigator software package (MBF Biosciences). Analyzed brain sections spanned from -1.22 to -3.52 mm from bregma, with every sixth slice used for quantification. The first section for each brain was randomly selected in order to avoid a sampling location bias. Ten-twelve sections were used for quantification from each animal. After delineation of the SGZ, or the granular layer of the DG for experiment group 3, at low magnification (20 × objective), the whole contour was imaged with 20–30 one-micron thick Z-stack images using a 63 × oil immersion objective (N.A. 1.4). Acquired images were first processed with Huygens deconvolution software (Scientific Volume Imaging) to improve resolution and signal to-noise ratio [26] and later processed offline using the optical dissector method. Section thickness was measured by focusing on the top of the section, setting the Z-axis to 0, and then refocusing to the bottom of the section and recording the actual thickness, at every counting location. Only clearly visible immunopositive cells co-labeled with an antibody against BrdU^+^ and either Neun, DCX or Sox2 (see Table 1), were counted. Additionally, cells were only counted if they did not intersect with the lines of exclusion on the counting grid. The total number of positive cell population was estimated in reference to the section volume and extrapolated for the total volume of the DG or hippocampus. The following parameters were set for cell counts: the counting frame was 140x104x15μm (height × width × dissector height), same size as the sampling grid for an exhaustive sampling regime of the hole contour, and a guard zone height of 2μm was used. For astrocytes estimation, the following parameters were set: the counting frame was 100x100x20μm (height × width × dissector height), the sampling grid size was defined as a mean of 25 sites and a guard zone height of 2μm. For neurons estimation, the following parameters were set: the counting frame was 30x30x15μm (height × width × dissector height), same size as the sampling grid for an exhaustive sampling regime of the hole contour, and a guard zone height of 2μm was used. These parameters were determined in a preliminary pilot study aimed at determining suitable counting frame and sampling grid parameters prior to counting (data available in supporting information file ‘S1 File’). An experimenter blind to all treatment groups performed the stereological counts. The coefficient of error (CE) Gunderson (m = 1) values was between 0.04–0.08 for all animals [27].

### Protein extraction from paraformaldehyde-fixed brain slices

Due to extensive molecular crosslinking in formalin-fixed tissues, an optimized method for protein extraction was utilized [28]. Hippocampal slices (n = 10–20, thickness = 40 μm) were placed in 50μl of lysis buffer (20mM tris–HCl containing 2% sodium dodecyl sulfate and 0.2M glycine at pH 9.0), boiled at 100°C for 20min followed by 2hr incubation at 80°C with agitation. Samples were then cooled on ice and centrifuged at 14,000g for 15 min and protein lysate was transferred to fresh tubes. Protein concentration was determined by BCA protein assay (Thermo scientific).

### Western blotting analysis of proteins

Protein lysates (15–40μg), in sample buffer (x5, 300mM Tris-HCl pH = 6.8, 10% SDS, 50% glycerol, 0.03% bromophenol blue, 500mM DTT), at a final volume of 15μl was loaded onto 10% Bis/Acrylamide gel and standard electrophoresis was performed. Separated proteins were blotted onto Polyvinylidene fluoride (PVDF, Invitrogen, USA) membranes using a wet blotter apparatus for 2 hours on 250mAmp on ice. The membrane was then blocked in 5% BSA for 1 hour at room temperature followed by overnight incubation with primary antibody (mAb Rabbit anti-Sirt6, (clone D8D12) Cat #12486, Cell Signaling). Blocking buffer and antibodies were prepared in 5% BSA. After incubation, membranes were washed 3x10 minutes in TBST, incubated with secondary antibody for 1 hour, washed again and finally incubated for 5 minutes with ECL. The membrane was then exposed with MicroChemi2 imager (DNR Bio-Imaging Systems, Israel).

### Data analysis and statistical analysis

All data presented are expressed as mean values ± S.E.M. Differences between normally distributed means were evaluated by a two-tailed Student's *t*-test for two group comparisons. Parametric two-way analysis of variance (ANOVA) with the Bonferroni *post-hoc* correction was performed to determine pairwise comparisons amongst multiple data sets. Statistical analysis was carried out using GraphPad Prism 5 software. For all tests, significance levels was set at *P* < 0.05.

## Results

### NPC proliferation is not altered by Sirt6 overexpression

**Fig 1:**
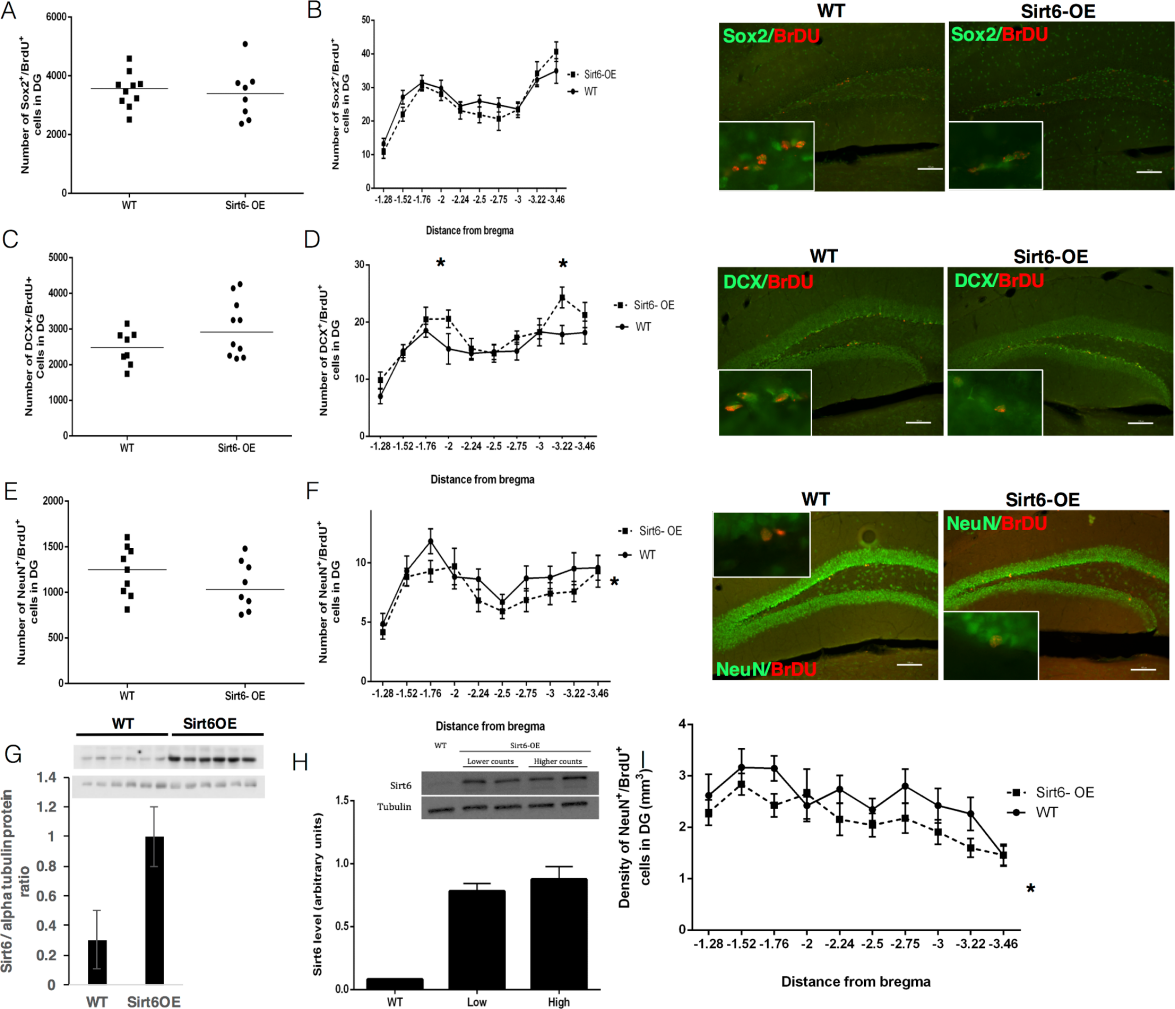
Sirt6-OE alters hippocampal neurogenesis. **(A)** Sox2^+^/BrdU^+^ cells were counted for each animal's SGZ in random bregma positions using the optical fractionator probe (Stereoinvestigator software, MBF bioscience), and total SGZ population estimation was extrapolated. Results were averaged across genotype group. No significant difference was found in total Sox2^+^/BrdU^+^ cells (t-test, p = 0.71). **(B)** Left panel: Distribution of Sox2^+^/BrdU^+^ neural progenitor cells in the SGZ of WT and Sirt6-OE mice. Results were averaged across genotype groups for each hippocampal slice according to distance from bregma position. No significant effect was found for genotype (2-way ANOVA, P = 0.14). Right panel: representative Sox2^+^/BrdU^+^ cell in the SGZ of WT and SIRT6-OE mice. **(C)** DCX^+^/BrdU^+^ cells were counted in the granular layer at random distances from bregma using an optical fractionator probe, and total SGZ population estimation was extrapolated for each animal. Results were averaged each across genotype. A higher, but not significant, number of total DCX^+^/BrdU^+^ cells were observed in Sirt6-OE mice compared to WT littermates (3022 ± 254.7 and 2469 ± 170.1 for WT and Sirt6-OE mice respectively, P = 0.1069, t-test). **(D)** Left panel: Distribution of DCX^+^/BrdU^+^ neural progenitor cells in the SGZ of WT and Sirt6-OE mice. Results were averaged across genotype group for each hippocampal slice according to distance from bregma. A significant genotype effect was found (2-way ANOVA, P = 0.005). Right panel: representative DCX^+^/BrdU^+^ cell in the SGZ of WT and SIRT6-OE mice. **(E)** NeuN^+^/BrdU^+^ cells were counted in the granular layer in random distances from bregma using an optical fractionator probe, and total SGZ population estimation was extrapolated for each animal. Results were averaged across genotype group. A lower, but not significant, number of total NeuN^+^/BrdU^+^ cells were observed in Sirt6-OE mice compared to WT littermates (1228 ± 90.68 and 1076 ± 95.36 for WT and Sirt6-OE mice respectively, P = 0.26, t-test). **(F)** Left panel: Number of total NeuN^+^/BrdU^+^ mature neurons in the SGZ of WT and Sirt6-OE mice. NeuN^+^/BrdU^+^ cells were counted in the granular layer in random distances from bregma in each animal using an optical fractionator probe. Results were averaged across genotype group for each hippocampal slice according to distance from bregma. A significant genotype effect was found (two-way ANOVA, F_(1,19)_ = 3.989, p = 0.018 for genotype effect). Right panel: representative NeuN^+^/BrdU^+^ cell in the SGZ of WT and SIRT6-OE mice. **(G)** Protein levels of WT and Sirt6-OE mice. Western blot analysis of WT and Sirt6-OE mice, blotted for SIRT6 and the house-keeping protein, α-tubulin. A robust increase in SIRT6 levels is clearly seen in Sirt6-OE mice. **()** Hippocampal SIRT6 protein levels from mice with high and low early neuron population numbers in the Sirt6-OE group. Upper panel. Western blot analysis of SIRT6 levels in Sirt6-OE mice with the highest and lowest new early neuron population counts, compared to a WT mouse with an average population count. Lower panel: Densitometric quantification of the corresponding bands was performed using ImageJ analysis software and each group was averaged. **(I)** NeuN^+^/BrdU^+^ cells were counted in the granular layer in random distances from bregma in each animal using an optical fractionator probe. Results were averaged across genotype for each bregma position and divided by the DG volume. A significant genotype effect was found (two-way ANOVA, F_(1,19)_ = 10.09, * p = 0.0017 for genotype main effect). Immunofluorescence images were taken at 10x magnification, scale bar = 20μm. Insets represent 63x magnification.

The neurogenic niche within the hippocampus contains dividing NPCs with a typical cell-cycle length of approximately 24 hours, with an S-phase of about 10 hours [1, 29]. In order to assess the rate of NPC proliferation in WT and Sirt6-OE mice, we counted the number of proliferating NPCs within a 24-hour time-frame. As BrdU has an *in-vivo* half-life of about 2 hours [30], we implemented a regimen of three BrdU pulses. One pulse of BrdU was injected every 8 hours to label all the dividing NPCs within the 24-hour time-frame (Table 1). To identify proliferating NPCs, we used the cell marker Sox2 [31] coupled with BrdU incorporation. Co-labeled cells in each genotype group were counted and averaged. No difference was found in the numbers of Sox2^+^/BrdU^+^ cells between genotype groups (P = 0.71, t-test, Fig 1A). To further investigate whether Sox2^+^/BrdU^+^ cells are evenly distributed throughout the hippocampus, we plotted the number of dividing NPCs throughout the rostro-caudal axis of the hippocampal SGZ. To do this, we used distance from bregma [24, 32] to normalize the location on the rostro-caudal axis of every examined brain slice. We observed a similar cellular distribution pattern between genotype groups, indicating no difference in the dynamics of NPC proliferation between the two strains of mice (Fig 1B, two-way ANOVA for genotype vs. bregma, p = 0.5643 for genotype main effect). Thus, this suggests that SIRT6 does not affect the cellular proliferation rate or distribution of NPCs in the DG.

### Sirt6 alters the distribution of differentiating DCX^+^/BrDU+ neural precursor cells

Doublecortin (DCX) is a cellular marker expressed on young neurons 1 to 20 days old following initiation of differentiation [33, 34]. We utilized DCX as a marker to assess whether SIRT6 alters the early differentiation of NPCs into neurons. The total number of DCX^+^/BrdU^+^ cells in the SGZ of Sirt6-OE mice was only moderately higher in Sirt6 OE mice compared with WT mice (3022 ± 254.7 and 2469 ± 170.1 respectively, P = 0.1069, t-test) (Fig 1C). To further investigate whether DCX^+^ cells are evenly distributed throughout the hippocampal SGZ, we plotted the number of DCX^+^/BrdU^+^ cells throughout the rostro-caudal axis of the hippocampal SGZ. This analysis yielded a significant main effect for genotype (Fig 1D, two-way ANOVA, F_(1,19)_ = 9.32, P = 0.005). This suggests that SIRT6 alters the distribution of neural precursors that are differentiating into neurons.

### Sirt6 decreases the number of mature NeuN+/BrDU+ neurons in the DG

We next tested whether Sirt6-OE alters the number of mature NeuN^+^ cells that survive the process of neurogenesis. To test this, cells co-expressing NeuN^+^/BrdU^+^ were counted throughout the DG. Sirt6-OE mice did not significantly alter the number of surviving mature neurons compared to their WT littermates (Fig 1E, 1228 ± 90.68 and 1076 ± 95.36 for WT and Sirt6-OE mice respectively, p = 0.26, t-test). However, the number of newly formed mature neurons throughout the rostral-caudal axis were significantly decreased (Fig 1F, two-way ANOVA, F_(1,19)_ = 3.989, P = 0.018 for genotype main effect). These results suggest that SIRT6 promotes the initiation of NPC differentiation toward a neuronal fate but restricts the final maturation of these newly formed neurons. Consistent with this, Sirt6-OE decreased the numbers of newly added NeuN^+^/BrdU^+^ cells in the DG granular layer (two-way ANOVA, F(_1,19_) = 10.09, P = 0.0017 for genotype main effect) (Fig 1I). This suggests that Sirt6-OE depresses the capability for network plasticity in these animals.

### Sirt6 overexpression levels are not correlated with DCX cell number

Compared with Brdu^+^/NeuN^+^, there was a large difference in the distribution of newly formed DCX^+^/BrdU^+^ cells between individual animals. Specifically, five Sirt6-OE mice exhibited higher DCX+/BrdU+ cell counts than all WT mice, whereas five Sirt6-OE mice exhibited population counts at the average levels of WT mice (Fig 1C). Thus, we examined whether differential over-expression of Sirt6 might account for the differences in the cell population counts. Sirt6-OE mice are homozygous for the transgene and express high levels of Sirt6 compared with heterozygous Sirt6-OE mice or control mice (Fig 1G). Thus, we compared the levels of SIRT6 in hippocampi from two mice with high DCX^+^/BrdU^+^ cell counts (4143.94 and 4260.00) with hippocampi from two mice which exhibited low DCX^+^/BrdU^+^ cell counts (2170.69 and 2198.00). Protein extracts from the hippocampi of these animals were comparable to WT mice, which exhibit a total 2261.25 DCX^+^/BrdU^+^ cells (Fig 1H). No correlation was observed between Sirt6 expression levels and the numbers of new early neurons in the hippocampus of Sirt6-OE mice. This suggests that there is a Sirt6 expression threshold that confers an effect on adult hippocampal neuronal differentiation and maturation in mice.

### Developmental Sirt6-OE does not alter gross anatomical hippocampal composition

**Fig 2:**
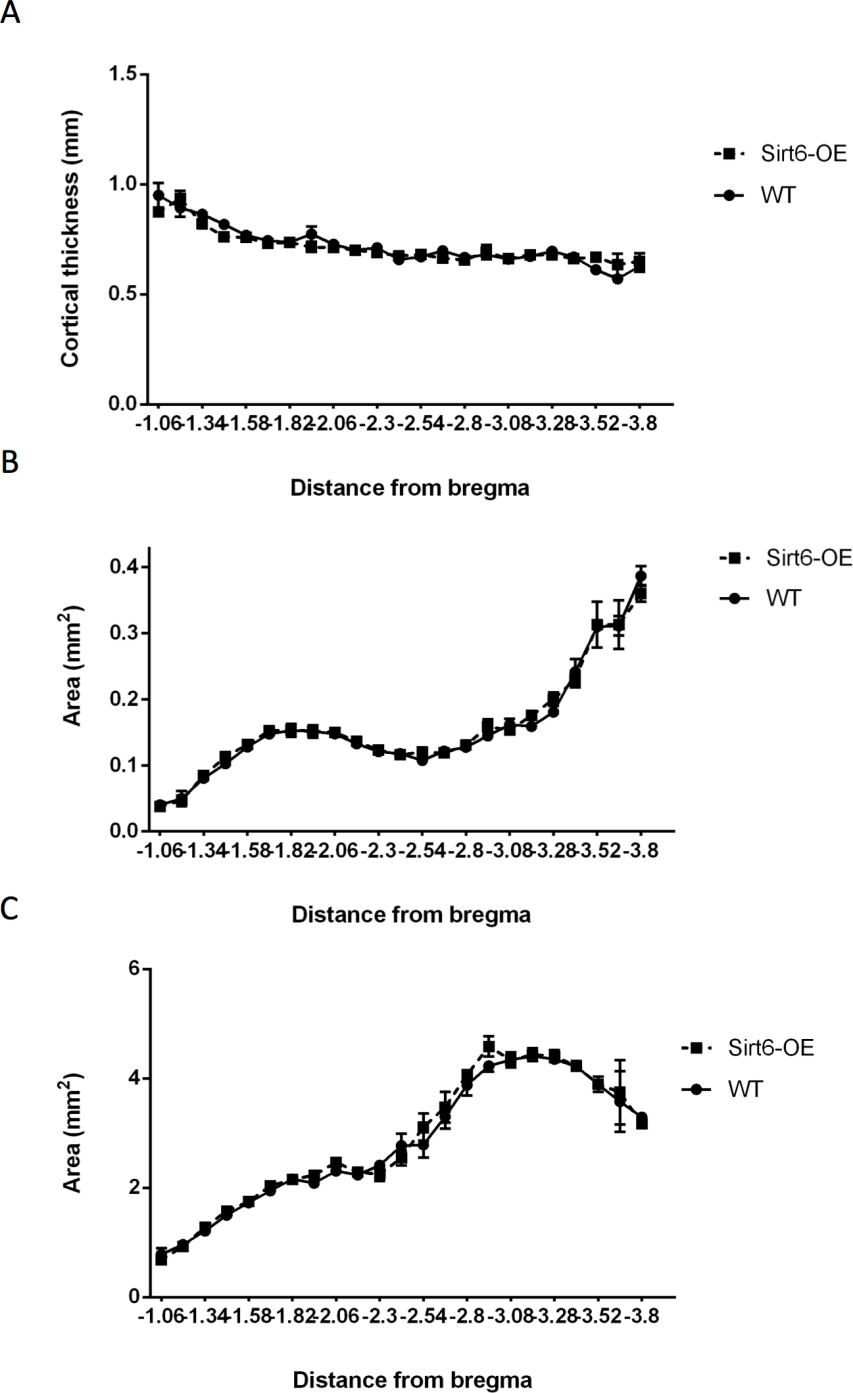
Cortical thickness of WT and Sirt6-OE mice. **(A)** Cortical thickness was measured perpendicular to the CA1 in the same location for each bregma investigated and averaged across genotype group. Measurements were made using Stereo investigator software (MBF bioscience). No significant difference was seen between the two groups (2-way ANOVA, p = 0.35). **(B)** Hippocampal area along the brain of WT and Sirt6-OE mice. Hippocampal area was measured by contouring the hippocampal slices for each bregma using Stereo investigator software (MBF bioscience) and averaging its surface area across genotype group. No significant difference was seen between the two groups (2-way ANOVA, P = 0.19). **(C)** Area of the dentate gyrus along the hippocampus of WT and Sirt6-OE mice. Dentate gyrus area was measured by contouring the DG region for each bregma using Stereo investigator software (MBF bioscience) and averaging its surface area across genotype group. No significant difference was seen between the two groups (2-way ANOVA, p = 0.48).

The developmental alterations of gene expression observed with Sirt6-OE mice could result in significant anatomical alterations in the brain. To address this, we examined basic brain anatomy, such as cortical thickness, hippocampal ultrastructure, and sub-hippocampal structures. No apparent structural differences were observed and no significant differences were seen between the two strains in cortical thickness (Fig 2A, two-way ANOVA, genotype main effect, p = 0.35), hippocampal volume (Fig 2B, two-way ANOVA, P = 0.19), and DG granular cell layer volume (Fig 2C, two-way ANOVA, P = 0.48). Thus, although Sirt6 is overexpressed during embryonic brain development, no gross anatomical abnormalities are observed.

### Sirt6-OE does not alter total glial or neuronal cell numbers in the hippocampus and cortex

**Fig 3:**
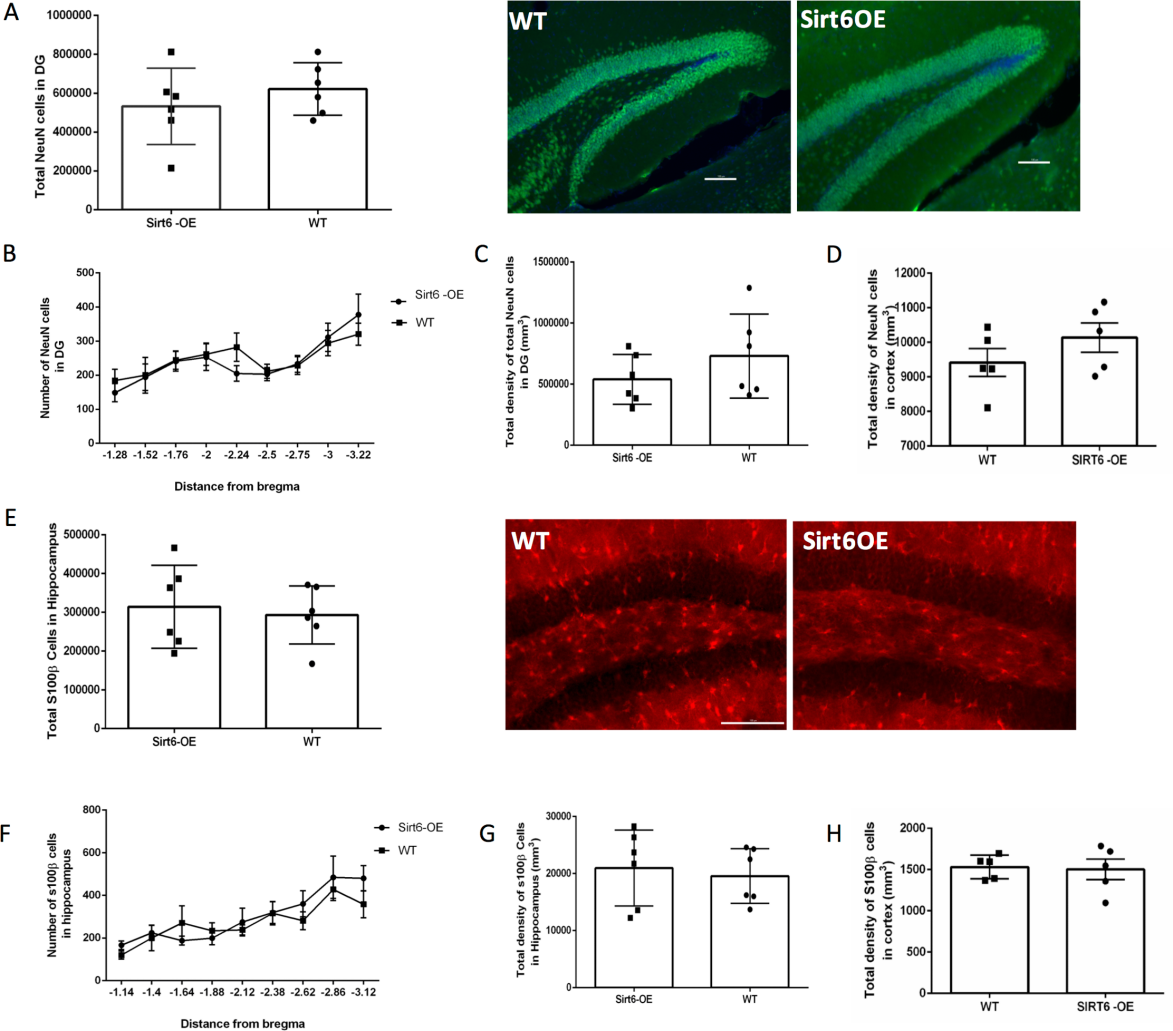
Sirt6-OE does not affect total numbers of DG and cortical NeuN^+^ or hippocampal and cortical S100β^+^ cells. **(A)** Left panel: Total numbers of NeuN^+^ cells were counted in the DG of WT (n = 6) and Sirt6-OE (n = 6) mice. Right panel: representative NeuN^+^ cells in the DG. Scale bar = 100μm **(B)** DG distribution of NeuN^+^ cells throughout the DG **(C)** Density of NeuN^+^ cells in the DG. (**D**) Density of NeuN^+^ cells in the cortex. **(E)** Left panel: Total numbers of S100β^+^ cells were counted in the hippocampi of WT (n = 6) and Sirt6-OE (n = 6) mice. Right panel: arrow indicates representative S100β^+^ cells in the hippocampus. Scale bar = 100μm **(F)** Hippocampal distribution of S100β^+^ cells. **(G)** Density of S100β^+^ cells in the hippocampus. () Density of S100β^+^ cells in the in the lateral parietal association cortex (LPtA) and primary somatosensory trunk cortex (S1Tr).

**Fig 4:**
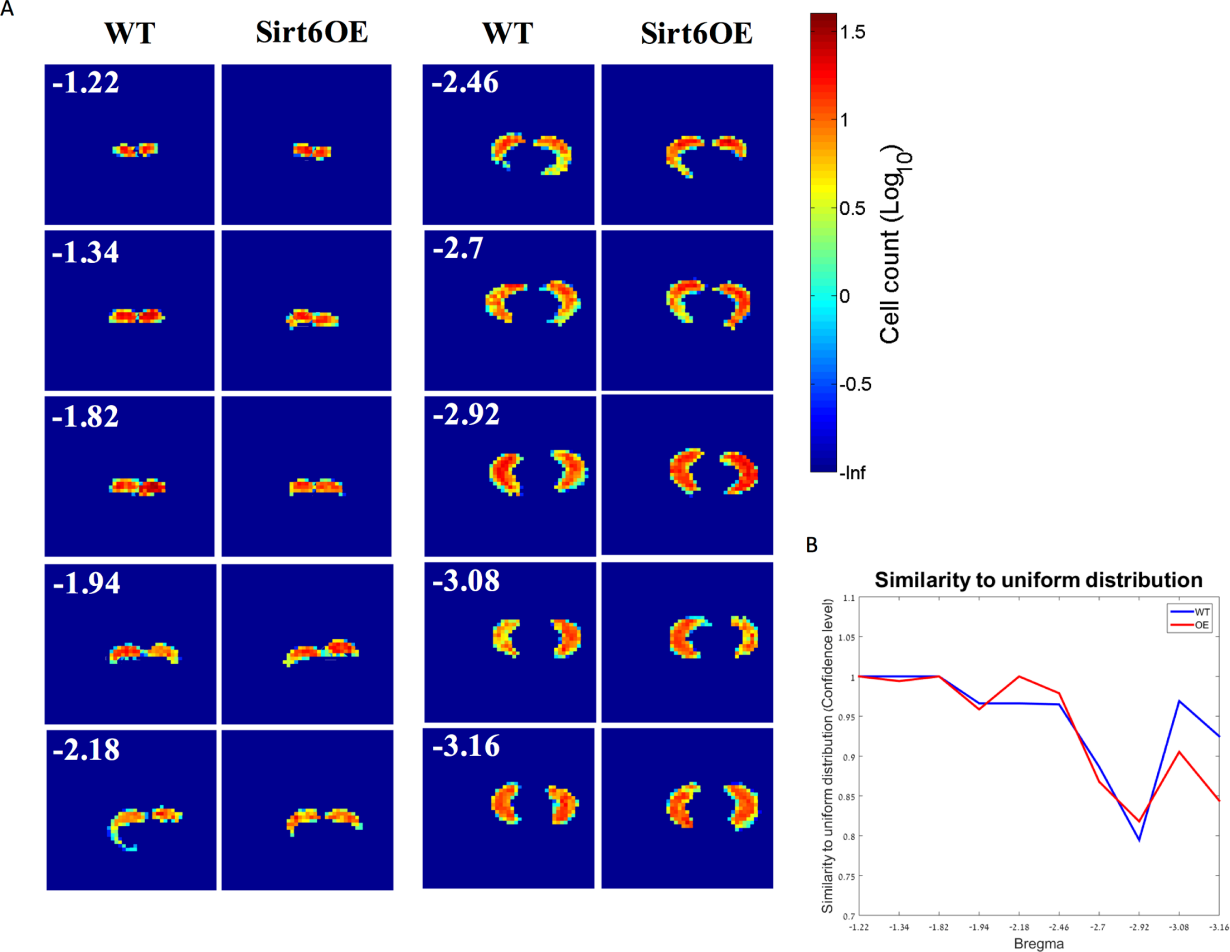
Sirt6-OE does not affect hippocampal distribution of astrocytes. Bivariate distribution of astrocytes scattering in the hippocampi of SIRT6 OE and WT mice. (A) Two dimensional scattering distributions of astrocytes in the following distances from bregma: -1.22, -1.34, -1.82, -1.94, -2.18, -2.46, -2.7, -2.92, -3.08, -3.16 of SIRT6 OE (*n* = 6) and WT (*n* = 6) hippocampi, logarithmic scale of cell count per square bin (*bin size* = 18^2^ μm). **(B)** Similarity of astrocyte distributions to a hypothetical uniform distribution of the same size and shape in the two-dimensional space as a function of bregma, expressed as the confidence level (1—P value), two-sample Kolmogorov-Smirnov test.

Embryonic neurogenesis occurs until embryonic (E) day 18 (E18) [35] and is replaced by gliogenesis which occurs until postnatal (P) day 2 (P2) [36] To assess whether developmental Sirt6-OE had a significant effect on the numbers of glial and neuronal cells in the adult hippocampus and cortex, we conducted counts of NeuN^+^ cells in the DG and the cortex, as well as counts of cells expressing S100ß^+^, an astrocytic marker, in the hippocampus and cortex. No significant differences were noted in total number (Fig 3A and 3B) or density of NeuN^+^ cells in the DG (Fig 3C) or in the cortex (Fig 3D). Similarly, no differences were observed in total number (Fig 3E and 3F) or density of S100β^+^ astrocytes in the hippocampus (Fig 3G) or cortex (Fig 3H). To further verify that both differentiation and migration of astrocytes are not impaired in Sirt6-OE mice, analyzed glial cell distribution. This analysis indicated that there is no significant difference between WT and Sirt6-OE mice in S100β^+^ cell distribution in the hippocampus (Fig 4A and 4B). These data suggest that developmental Sirt6-OE does not confer significant effects on the total number of neurons and astrocytes in the hippocampal and cortical regions analyzed.

## Discussion

About 3000–4000 new potential neurons are estimated to be born every day in the rodent hippocampus [37]. Of those, less than 50% differentiate, mature, and survive to adulthood [38, 39]. Unlike in the embryonic brain, where neurogenesis is a transient phenomenon, the adult rodent SGZ is thought to retain neurogenic capacity for the entire life of the animal [6]. Aging in rodents results in a continuous decline in adult NPCs and neurogenesis, which is correlated with impaired cognitive function [6]. The longevity gene, Sirt6, affects cellular maintenance, stress protection [40] and regulates cellular differentiation [17–19]. Sirt6 is also expressed in the hippocampus [13], and our data suggest that SIRT6 also regulates adult hippocampal neurogenesis.

Few studies have examined the impact of SIRT6 on cellular differentiation. SIRT6 positively regulates the differentiation of rat bone marrow mesenchymal stem cells [18], proliferation and differentiation of chondrocytes [19], and differentiation of vascular smooth muscle cells in response to cyclic strain [17]. In addition, SIRT6 potentially affects embryonic stem cell (ESC) differentiation by regulating levels of H3K56ac and H3K9ac on pluripotent genes [41]. Moreover, both SIRT2 and SIRT6 also contribute to post ischemia neurogenesis [42]. Interestingly, in our experiments, Sirt6-OE did not alter NPC proliferation in vivo, but increased the proportions of young neurons while decreasing the proportions of mature neurons. This suggests that SIRT6 is one of multiple key players that regulate the complexity of neurogenesis.

Neurogenesis is facilitated by several regulatory pathways that are activated at different stages of differentiation [43]. Genes which serve a crucial role in one stage may exert other effects in a different stage, unless strictly regulated according to the cellular differentiation scheme. Our results indicate that Sirt6-OE differentially regulates different stages of neurogenesis. This is consistent with previous reports, which showed that different aspects of adult hippocampal neurogenesis (i.e. proliferation, differentiation and survival) are differentially affected by the genetic background of mice [38, 44, 45]. For example, p50-deficient mice exhibit lower levels of neurogenesis that were not manifested by differences in the number of immature neurons [46]. Interestingly, SIRT6 regulates the action of NF-κB, a heterodimeric complex of p50 (encoded by nuclear factor-kappa B (NF-κB)) and p65 (encoded by RelA). SIRT6 is physically present at promoters of genes activated by NF-κB, and by docking with RelA/p65 deacetylates histone H3 lysine 9 (H3K9) to repress the transcription of NF-κB target genes [47]. This regulation of NF-kB might account for the negative effect of SIRT6 on final neuronal maturation. In contrast, SIRT6 is known to facilitate growth plate chondrocyte differentiation through regulation of sonic hedgehog (Shh) signaling pathway genes [19]. Thus, increased neuronal differentiation in Sirt6-OE mice might be the outcome of shh signaling, which is known to be crucial for neural differentiation [48, 49]. Because we found no correlation between SIRT6 expression levels and the number of newly differentiated neurons in Sirt6-OE mice (Fig 1H), we suggest that the effects of Sirt6-OE on NPC differentiation and maturation in the DG are related to downstream effects of SIRT6 rather than SIRT6 expression levels per-se. Quantification of total NeuN+ cells in the DG and cortex as well as S100β^+^ cells in the hippocampus and cortex indicate no significant difference compared with WT mice. Thus, the observed effects of Sirt6-OE on neuronal differentiation are likely to be restricted to adult hippocampal neuronal differentiation and maturation in mice, although conclusions regarding effects on embryonic neurogenesis and gliogenesis cannot be drawn.

SIRT1 and SIRT6 are both nuclear sirtuins, with shared targets and functions [5, 47, 50–53]. SIRT1 regulates the restricted differentiation of embryonic and mesenchymal stem cells [54, 55]. Hisahara et al. showed that when NPCs are differentiated in culture, SIRT1 immediately translocates to the nucleus. Reducing the expression of SIRT1 suppresses NPC differentiation toward a neuronal fate without affecting differentiation rates of glial cells [56]. However, Prozorovski and colleagues found that under mild oxidative stress conditions, SIRT1 interacts with Hes1 to repress MASH1 transcription, resulting in NPC differentiation to astrocytes rather than neurons [2]. Hence, SIRT1 may exhibit opposite effects under different cellular conditions. Given the roles of SIRT6 in cellular maintenance, these findings may imply a more pronounced effect of SIRT6 on neurogenesis under physiological stressors such as oxidative stress, calorie restriction, physical exercise or aging.

In this study, we used transgenic mice on a C57bl/6 background in which hippocampal Sirt6 expression levels were elevated. A constitutive OE of Sirt6 raises the question of developmental effects related to early expression of the transgene during embryonic development, which may account for the difference in the neural network regeneration process, regardless of SIRT6 action. Our *in-vivo* observations indicate that no gross anatomical differences were observed between the brains of Sirt6-OE and WT mice, and no difference in the number of NeuN^+^ cells in the adult DG and cortex or S100β^+^ cells in the hippocampus and cortex of Sirt6 overexpressing mice compared with WT mice. Thus, while we observed an effect of Sirt6 on adult hippocampal neuronal differentiation and maturation in mice, we cannot entirely rule out effects on embryonic neurogenesis and gliogenesis.

In conclusion, this data offers a new perspective on the function of SIRT6 in the nervous system. The dual effect of increased neural differentiation along with a decreased turnover of new neurons generated in Sirt6-OE mouse hippocampi, adds to our understanding of the complexity of adult hippocampal neurogenesis.

## Supporting information

S1 File Stereology parameters were determined in a preliminary pilot study aimed at determining suitable counting frame and sampling grid parameters prior to counting (data available in supporting information file ‘S1 File’). (XLSX)

## References

[1] Ming GL, Song. Adult neurogenesis in the mammalian brain: significant answers and significant questions. Neuron. 2011;70(4):687–702. doi: 10.1016/j.neuron.2011.05.001 ; PubMed Central PMCID: PMCPMC3106107. 21609825 10.1016/j.neuron.2011.05.001 PMC3106107

[2] Prozorovski T, Schulze-Topphoff U, Glumm R, Baumgart J, Schröter F, Ninnemann O, et al Sirt1 contributes critically to the redox-dependent fate of neural progenitors. Nat Cell Biol. 2008;10(4):385–94. doi: 10.1038/ncb1700 . 18344989 10.1038/ncb1700

[3] Kumar V, Pandey A, Jahan S, Shukla RK, Kumar D, Srivastava A, et al Differential responses of Trans-Resveratrol on proliferation of neural progenitor cells and aged rat hippocampal neurogenesis. Sci Rep. 2016;6:28142 doi: 10.1038/srep28142 . 27334554 10.1038/srep28142 PMC4917886

[4] Ma CY, Yao MJ, Zhai QW, Jiao JW, Yuan XB, Poo MM. SIRT1 suppresses self-renewal of adult hippocampal neural stem cells. Development. 2014;141(24):4697–709. doi: 10.1242/dev.117937 . 25468938 10.1242/dev.117937

[5] Kanfi, Naiman S, Amir G, Peshti V, Zinman G, Nahum L, et al The sirtuin SIRT6 regulates lifespan in male mice. Nature. 2012;483(7388):218–21. doi: 10.1038/nature10815 . 22367546 10.1038/nature10815

[6] van Praag, Shubert T, Zhao C, Gage FH. Exercise enhances learning and hippocampal neurogenesis in aged mice. J Neurosci. 2005;25(38):8680–5. doi: 10.1523/JNEUROSCI.1731-05.2005 ; PubMed Central PMCID: PMCPMC1360197. 16177036 10.1523/JNEUROSCI.1731-05.2005 PMC1360197

[7] Bondolfi L, Ermini F, Long JM, Ingram DK, Jucker M. Impact of age and caloric restriction on neurogenesis in the dentate gyrus of C57BL/6 mice. Neurobiol Aging. 2004;25(3):333–40. doi: 10.1016/S0197-4580(03)00083-6 . 15123339 10.1016/S0197-4580(03)00083-6

[8] Carro E, Trejo JL, Busiguina S, Torres-Aleman I. Circulating insulin-like growth factor I mediates the protective effects of physical exercise against brain insults of different etiology and anatomy. J Neurosci. 2001;21(15):5678–84. . 11466439 10.1523/JNEUROSCI.21-15-05678.2001 PMC6762673

[9] Cantó C, Auwerx J. Calorie restriction: is AMPK a key sensor and effector? Physiology (Bethesda). 2011;26(4):214–24. doi: 10.1152/physiol.00010.2011 ; PubMed Central PMCID: PMCPMC3627048. 21841070 10.1152/physiol.00010.2011 PMC3627048

[10] Richter EA, Ruderman NB. AMPK and the biochemistry of exercise: implications for human health and disease. Biochem J. 2009;418(2):261–75. doi: 10.1042/BJ20082055 ; PubMed Central PMCID: PMCPMC2779044. 19196246 10.1042/BJ20082055 PMC2779044

[11] Imai S. The NAD World: a new systemic regulatory network for metabolism and aging—Sirt1, systemic NAD biosynthesis, and their importance. Cell Biochem Biophys. 2009;53(2):65–74. doi: 10.1007/s12013-008-9041-4 ; PubMed Central PMCID: PMCPMC2734380. 19130305 10.1007/s12013-008-9041-4 PMC2734380

[12] Maalouf M, Rho JM, Mattson MP. The neuroprotective properties of calorie restriction, the ketogenic diet, and ketone bodies. Brain Res Rev. 2009;59(2):293–315. doi: 10.1016/j.brainresrev.2008.09.002 ; PubMed Central PMCID: PMCPMC2649682. 18845187 10.1016/j.brainresrev.2008.09.002 PMC2649682

[13] Schwer B, Schumacher B, Lombard DB, Xiao C, Kurtev MV, Gao J, et al Neural sirtuin 6 (Sirt6) ablation attenuates somatic growth and causes obesity. Proc Natl Acad Sci U S A. 2010;107(50):21790–4. doi: 10.1073/pnas.1016306107 ; PubMed Central PMCID: PMCPMC3003110. 21098266 10.1073/pnas.1016306107 PMC3003110

[14] Yang SJ, Choi JM, Chae SW, Kim WJ, Park SE, Rhee EJ, et al Activation of peroxisome proliferator-activated receptor gamma by rosiglitazone increases sirt6 expression and ameliorates hepatic steatosis in rats. PLoS One. 2011;6(2):e17057 doi: 10.1371/journal.pone.0017057 ; PubMed Central PMCID: PMCPMC3044155. 21373642 10.1371/journal.pone.0017057 PMC3044155

[15] Elhanati S, Kanfi, Varvak A, Roichman A, Carmel-Gross I, Barth S, et al Multiple regulatory layers of SREBP1/2 by SIRT6. Cell Rep. 2013;4(5):905–12. doi: 10.1016/j.celrep.2013.08.006 . 24012758 10.1016/j.celrep.2013.08.006

[16] Mostoslavsky R, Chua KF, Lombard DB, Pang, Fischer MR, Gellon L, et al Genomic instability and aging-like phenotype in the absence of mammalian SIRT6. Cell. 2006;124(2):315–29. doi: 10.1016/j.cell.2005.11.044 . 16439206 10.1016/j.cell.2005.11.044

[17] Yao QP, Zhang P, Qi YX, Chen SG, Shen BR, Han, et al The role of SIRT6 in the differentiation of vascular smooth muscle cells in response to cyclic strain. Int J Biochem Cell Biol. 2014 doi: 10.1016/j.biocel.2014.01.016 . 24495875 10.1016/j.biocel.2014.01.016

[18] Sun Wu, Fu D Liu, Huang C. SIRT6 regulates osteogenic differentiation of rat bone marrow mesenchymal stem cells partially via suppressing the nuclear factor-kappa B signaling pathway. Stem Cells. 2014 doi: 10.1002/stem.1671 . 24510807 10.1002/stem.1671

[19] Piao J, Tsuji K, Ochi, Iwata M, Koga D, Okawa A, et al Sirt6 regulates postnatal growth plate differentiation and proliferation via Ihh signaling. Sci Rep. 2013;3:3022 doi: 10.1038/srep03022 . 24149372 10.1038/srep03022 PMC6505680

[20] Niwa, Yamamura K, Miyazaki J. Efficient selection for high-expression transfectants with a novel eukaryotic vector. Gene. 1991;108(2):193–9. . 1660837 10.1016/0378-1119(91)90434-d

[21] Kanfi, Peshti V, Gil R, Naiman S, Nahum L, Levin E, et al SIRT6 protects against pathological damage caused by diet-induced obesity. Aging Cell. 2010;9(2):162–73. doi: 10.1111/j.1474-9726.2009.00544.x . 20047575 10.1111/j.1474-9726.2009.00544.x

[22] Zhao C, Deng, Gage FH. Mechanisms and functional implications of adult neurogenesis. Cell. 2008;132(4):645–60. doi: 10.1016/j.cell.2008.01.033 . 18295581 10.1016/j.cell.2008.01.033

[23] Gage GJ, Kipke DR, Shain. Whole animal perfusion fixation for rodents. J Vis Exp. 2012;(65). doi: 10.3791/3564 ; PubMed Central PMCID: PMCPMC3476408. 22871843 10.3791/3564 PMC3476408

[24] Franklin KBJ, Paxinos G. <<The>> mouse brain in stereotaxic coordinates Compact 3rd ed. Amsterdam: Elsevier Academic Press; 2008 xxx, 242 p. (spiral) p.

[25] West MJ, Slomianka L, Gundersen HJ. Unbiased stereological estimation of the total number of neurons in thesubdivisions of the rat hippocampus using the optical fractionator. Anat Rec. 1991;231(4):482–97. doi: 10.1002/ar.1092310411 . 1793176 10.1002/ar.1092310411

[26] McNally JG, Karpova T, Cooper J, Conchello JA. Three-dimensional imaging by deconvolution microscopy. Methods. 1999;19(3):373–85. doi: 10.1006/meth.1999.0873 . 10579932 10.1006/meth.1999.0873

[27] Gundersen HJ, Jensen EB, Kiêu K, Nielsen J. The efficiency of systematic sampling in stereology—reconsidered. J Microsc. 1999;193(Pt 3):199–211. . 10348656 10.1046/j.1365-2818.1999.00457.x

[28] Guo Liu, Ju Z, Tamboli P, Jonasch E, Mills GB, et al An efficient procedure for protein extraction from formalin-fixed, paraffin-embedded tissues for reverse phase protein arrays. Proteome Sci. 2012;10(1):56 doi: 10.1186/1477-5956-10-56 ; PubMed Central PMCID: PMCPMC3561137. 23006314 10.1186/1477-5956-10-56 PMC3561137

[29] Brandt MD, Hübner M, Storch A. Brief report: Adult hippocampal precursor cells shorten S-phase and total cell cycle length during neuronal differentiation. Stem Cells. 2012;30(12):2843–7. doi: 10.1002/stem.1244 . 22987479 10.1002/stem.1244

[30] Deng, Aimone JB, Gage FH. New neurons and new memories: how does adult hippocampal neurogenesis affect learning and memory? Nat Rev Neurosci. 2010;11(5):339–50. doi: 10.1038/nrn2822 ; PubMed Central PMCID: PMCPMC2886712. 20354534 10.1038/nrn2822 PMC2886712

[31] Lee KE, Seo J, Shin J, Ji EH, Roh J, Kim JY, et al Positive feedback loop between Sox2 and Sox6 inhibits neuronal differentiation in the developing central nervous system. Proc Natl Acad Sci U S A. 2014 doi: 10.1073/pnas.1308758111 . 24501124 10.1073/pnas.1308758111 PMC3932859

[32] Donoghue JF, Bogler O, Johns TG. A simple guide screw method for intracranial xenograft studies in mice. J Vis Exp. 2011;(55). doi: 10.3791/3157 ; PubMed Central PMCID: PMCPMC3230180. 21968439 10.3791/3157 PMC3230180

[33] Klempin F, Marr RA, Peterson DA. Modification of pax6 and olig2 expression in adult hippocampal neurogenesis selectively induces stem cell fate and alters both neuronal and glial populations. Stem Cells. 2012;30(3):500–9. doi: 10.1002/stem.1005 ; PubMed Central PMCID: PMCPMC3635101. 22162276 10.1002/stem.1005 PMC3635101

[34] Plümpe T, Ehninger D, Steiner B, Klempin F, Jessberger S, Brandt M, et al Variability of doublecortin-associated dendrite maturation in adult hippocampal neurogenesis is independent of the regulation of precursor cell proliferation. BMC Neurosci. 2006;7:77 doi: 10.1186/1471-2202-7-77 ; PubMed Central PMCID: PMCPMC1657022. 17105671 10.1186/1471-2202-7-77 PMC1657022

[35] Miller FD, Gauthier AS. Timing is everything: making neurons versus glia in the developing cortex. Neuron. 2007;54(3):357–69. doi: 10.1016/j.neuron.2007.04.019 . 17481390 10.1016/j.neuron.2007.04.019

[36] Qian X, Shen Q, Goderie SK, He, Capela A, Davis AA, et al Timing of CNS cell generation: a programmed sequence of neuron and glial cell production from isolated murine cortical stem cells. Neuron. 2000;28(1):69–80. . 11086984 10.1016/s0896-6273(00)00086-6

[37] Hayes NL, Nowakowski RS. Dynamics of cell proliferation in the adult dentate gyrus of two inbred strains of mice. Brain Res Dev Brain Res. 2002;134(1–2):77–85. . 11947938 10.1016/s0165-3806(01)00324-8

[38] Kempermann G, Kuhn HG, Gage FH. Genetic influence on neurogenesis in the dentate gyrus of adult mice. Proc Natl Acad Sci U S A. 1997;94(19):10409–14. ; PubMed Central PMCID: PMCPMC23376. 9294224 10.1073/pnas.94.19.10409 PMC23376

[39] van Praag, Kempermann G, Gage FH. Running increases cell proliferation and neurogenesis in the adult mouse dentate gyrus. Nat Neurosci. 1999;2(3):266–70. doi: 10.1038/6368 . 10195220 10.1038/6368

[40] Simeoni F, Tasselli L, Tanaka S, Villanova L, Hayashi M, Kubota K, et al Proteomic analysis of the SIRT6 interactome: novel links to genome maintenance and cellular stress signaling. Sci Rep. 2013;3:3085 doi: 10.1038/srep03085 ; PubMed Central PMCID: PMCPMC3812651. 24169447 10.1038/srep03085 PMC3812651

[41] Etchegaray JP, Chavez L, Huang, Ross KN, Choi J, Martinez-Pastor B, et al The histone deacetylase SIRT6 controls embryonic stem cell fate via TET-mediated production of 5-hydroxymethylcytosine. Nat Cell Biol. 2015;17(5):545–57. doi: 10.1038/ncb3147 ; PubMed Central PMCID: PMCPMC4593707. 25915124 10.1038/ncb3147 PMC4593707

[42] Zhao, Guan YF, Zhou XM, Li GQ, Li ZY, Zhou CC, et al Regenerative Neurogenesis After Ischemic Stroke Promoted by Nicotinamide Phosphoribosyltransferase-Nicotinamide Adenine Dinucleotide Cascade. Stroke. 2015;46(7):1966–74. doi: 10.1161/STROKEAHA.115.009216 . 26060246 10.1161/STROKEAHA.115.009216

[43] Faigle R, Song. Signaling mechanisms regulating adult neural stem cells and neurogenesis. Biochim Biophys Acta. 2013;1830(2):2435–48. doi: 10.1016/j.bbagen.2012.09.002 ; PubMed Central PMCID: PMCPMC3541438. 22982587 10.1016/j.bbagen.2012.09.002 PMC3541438

[44] Steele AD, Emsley JG, Ozdinler PH, Lindquist S, Macklis JD. Prion protein (PrPc) positively regulates neural precursor proliferation during developmental and adult mammalian neurogenesis. Proc Natl Acad Sci U S A. 2006;103(9):3416–21. doi: 10.1073/pnas.0511290103 ; PubMed Central PMCID: PMCPMC1413927. 16492732 10.1073/pnas.0511290103 PMC1413927

[45] Matsumata M, Sakayori N, Maekawa M, Owada, Yoshikawa T, Osumi N. The effects of Fabp7 and Fabp5 on postnatal hippocampal neurogenesis in the mouse. Stem Cells. 2012;30(7):1532–43. doi: 10.1002/stem.1124 . 22581784 10.1002/stem.1124

[46] Denis-Donini S, Dellarole A, Crociara P, Francese MT, Bortolotto V, Quadrato G, et al Impaired adult neurogenesis associated with short-term memory defects in NF-kappaB p50-deficient mice. J Neurosci. 2008;28(15):3911–9. doi: 10.1523/JNEUROSCI.0148-08.2008 . 18400889 10.1523/JNEUROSCI.0148-08.2008 PMC6670458

[47] Kawahara TL, Michishita E, Adler AS, Damian M, Berber E, Lin M, et al SIRT6 links histone H3 lysine 9 deacetylation to NF-kappaB-dependent gene expression and organismal life span. Cell. 2009;136(1):62–74. doi: 10.1016/j.cell.2008.10.052 ; PubMed Central PMCID: PMCPMC2757125. 19135889 10.1016/j.cell.2008.10.052 PMC2757125

[48] Ericson J, Muhr J, Placzek M, Lints T, Jessell TM, Edlund T. Sonic hedgehog induces the differentiation of ventral forebrain neurons: a common signal for ventral patterning within the neural tube. Cell. 1995;81(5):747–56. . 7774016 10.1016/0092-8674(95)90536-7

[49] Hynes M, Porter JA, Chiang C, Chang D, Tessier-Lavigne M, Beachy PA, et al Induction of midbrain dopaminergic neurons by Sonic hedgehog. Neuron. 1995;15(1):35–44. . 7619528 10.1016/0896-6273(95)90062-4

[50] Michishita E, McCord RA, Berber E, Kioi M, Padilla-Nash, Damian M, et al SIRT6 is a histone H3 lysine 9 deacetylase that modulates telomeric chromatin. Nature. 2008;452(7186):492–6. doi: 10.1038/nature06736 ; PubMed Central PMCID: PMCPMC2646112. 18337721 10.1038/nature06736 PMC2646112

[51] Vaquero A, Scher M, Lee D, Erdjument-Bromage, Tempst P, Reinberg D. Human SirT1 interacts with histone H1 and promotes formation of facultative heterochromatin. Mol Cell. 2004;16(1):93–105. doi: 10.1016/j.molcel.2004.08.031 . 15469825 10.1016/j.molcel.2004.08.031

[52] Yeung F, Hoberg JE, Ramsey CS, Keller MD, Jones DR, Frye RA, et al Modulation of NF-kappaB-dependent transcription and cell survival by the SIRT1 deacetylase. EMBO J. 2004;23(12):2369–80. doi: 10.1038/sj.emboj.7600244 ; PubMed Central PMCID: PMCPMC423286. 15152190 10.1038/sj.emboj.7600244 PMC423286

[53] Satoh A, Brace CS, Rensing N, Cliften P, Wozniak DF, Herzog ED, et al Sirt1 extends life span and delays aging in mice through the regulation of Nk2 homeobox 1 in the DMH and LH. Cell Metab. 2013;18(3):416–30. doi: 10.1016/j.cmet.2013.07.013 ; PubMed Central PMCID: PMCPMC3794712. 24011076 10.1016/j.cmet.2013.07.013 PMC3794712

[54] Simic P, Zainabadi K, Bell E, Sykes DB, Saez B, Lotinun S, et al SIRT1 regulates differentiation of mesenchymal stem cells by deacetylating β-catenin. EMBO Mol Med. 2013;5(3):430–40. doi: 10.1002/emmm.201201606 ; PubMed Central PMCID: PMCPMC3598082. 23364955 10.1002/emmm.201201606 PMC3598082

[55] Calvanese V, Lara E, Suárez-Alvarez B, Abu Dawud R, Vázquez-Chantada M, Martínez-Chantar ML, et al Sirtuin 1 regulation of developmental genes during differentiation of stem cells. Proc Natl Acad Sci U S A. 2010;107(31):13736–41. doi: 10.1073/pnas.1001399107 ; PubMed Central PMCID: PMCPMC2922228. 20631301 10.1073/pnas.1001399107 PMC2922228

[56] Hisahara S, Chiba S, Matsumoto, Tanno M, Yagi, Shimohama S, et al Histone deacetylase SIRT1 modulates neuronal differentiation by its nuclear translocation. Proc Natl Acad Sci U S A. 2008;105(40):15599–604. doi: 10.1073/pnas.0800612105 ; PubMed Central PMCID: PMCPMC2563076. 18829436 10.1073/pnas.0800612105 PMC2563076

